# Supplementary material for: Adaptive translational reprogramming of metabolism limits the response to targeted therapy in BRAFV600 melanoma
Source: Nat Commun. 2022 Mar 1;13:1100. doi: 10.1038/s41467-022-28705-x (PMC8888590; doi:10.1038/s41467-022-28705-x)
Supplement: Supplementary file 1 — Supplementary Information [file 41467_2022_28705_MOESM1_ESM.pdf]

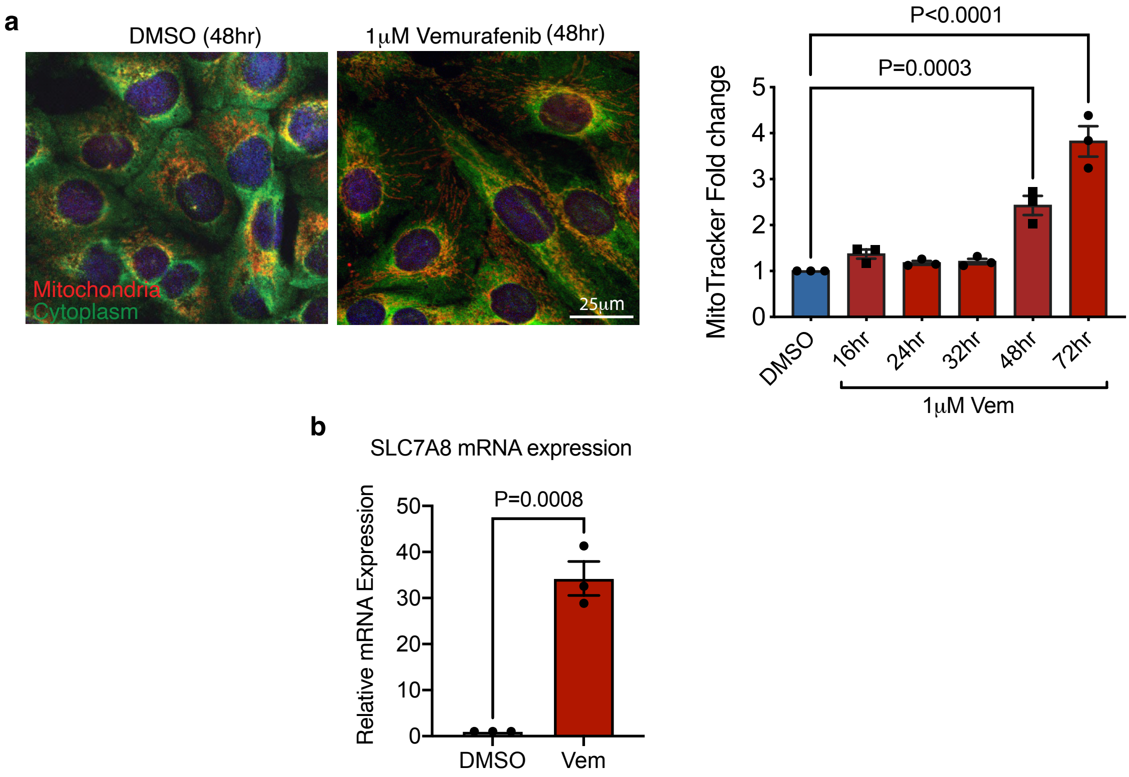

Supplementary Figure 1. Functional characterization of adaptive metabolic reprogramming following BRAF inhibition in BRAF^{V600} melanoma cells

A. A375 cells were treated with 1μM Vem for the indicated time and assessed for mitochondrial number using the mitochondrial stain MitoTracker. Representative confocal images of n=3 biologically independent experiments are shown (left panel) and quantitation was performed using high content image analysis (right panel). Data are presented as mean fold change relative to DMSO control, +/- SEM; n=3 biologically independent experiments. Statistical significance was determined using a one-way ANOVA adjusted for multiple comparisons. **B.** Gene expression was determined using q-RT-PCR. Data are presented as mean fold change relative to DMSO control, +/- SEM; n=3 biologically independent experiments. Statistical significance was determined using a two tailed Students T test. SLC7A8 = Solute Carrier Family 7 Member 8. Source data are provided as a source data file.

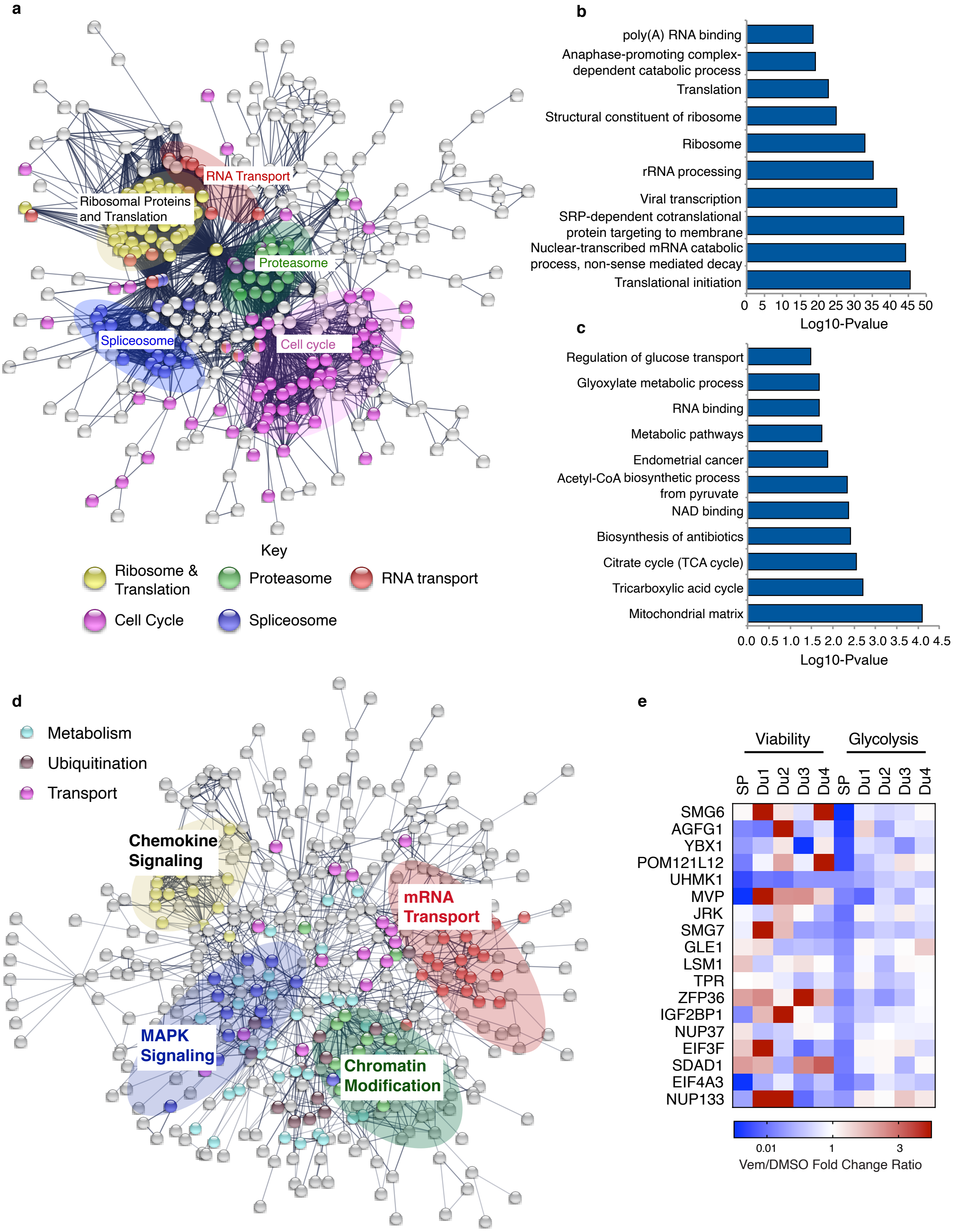
a
RNA Transport
Ribosomal Proteins and Translation
Proteasome
Spliceosome
Cell cycle
Key
Ribosome & Translation
Proteasome
RNA transport
Cell Cycle
Spliceosome
b
poly(A) RNA binding
Anaphase-promoting complex-dependent catabolic process
Translation
Structural constituent of ribosome
Ribosome
rRNA processing
Viral transcription
SRP-dependent cotranslational protein targeting to membrane
Nuclear-transcribed mRNA catabolic process, non-sense mediated decay
Translational initiation
0 5 10 15 20 25 30 35 40 45 50
Log10-Pvalue
c
Regulation of glucose transport
Glyoxylate metabolic process
RNA binding
Metabolic pathways
Endometrial cancer
Acetyl-CoA biosynthetic process from pyruvate
NAD binding
Biosynthesis of antibiotics
Citrate cycle (TCA cycle)
Tricarboxylic acid cycle
Mitochondrial matrix
0.0 0.5 1.0 1.5 2.0 2.5 3.0 3.5 4.0 4.5
Log10-Pvalue
d
Metabolism
Ubiquitination
Transport
Chemokine Signaling
mRNA Transport
MAPK Signaling
Chromatin Modification
e
Viability
Glycolysis
SP Du1 Du2 Du3 Du4 SP Du1 Du2 Du3 Du4
SMG6
AGFG1
YBX1
POM121L12
UHMK1
MVP
JRK
SMG7
GLE1
LSM1
TPR
ZFP36
IGF2BP1
NUP37
EIF3F
SDAD1
EIF4A3
NUP133
0.01
1
3
Vem/DMSO Fold Change Ratio

Supplementary Figure 2. A genome wide screen for regulators of glycolytic responses to BRAF inhibition

A. Network analysis was performed on the 622 viability screen hits (DMSO ΔT48 > -1.5 Z-score; Source data are provided in Supplementary Data 1) using String. Enriched pathways were mapped onto the network as indicated. **B.** Functional annotation enrichment analysis was performed on the viability screen hits using DAVID. **C.** Functional annotation enrichment analysis was performed on the 164 glycolysis screen hits (DMSO lactate per cell ratio > 0.5-fold change; Source data are provided in Supplementary Data 2) using DAVID. **D.** Network analysis was performed on the 717 genes that enhanced the effects of Vem on lactate production (DMSO and Vem ΔT48 cell count > 0.3-fold change; DMSO lactate per cell ratio > 0.4-fold change; and Vem lactate per cell ratio < 0.5-fold change; Supplementary Data 3) using String. Enriched pathways were mapped onto the network as indicated. **E.** Viability and glycolysis data from the secondary validation screen for genes associated with RNA binding, transport and translation. Individual siRNA duplexes (Du1-4) comprising each SMARTpool (SP) were arrayed into individual wells to confirm reproducibility of phenotypes. Data is expressed as Vem/DMSO fold change ratio. Source data for E are provided as a Source Data file.

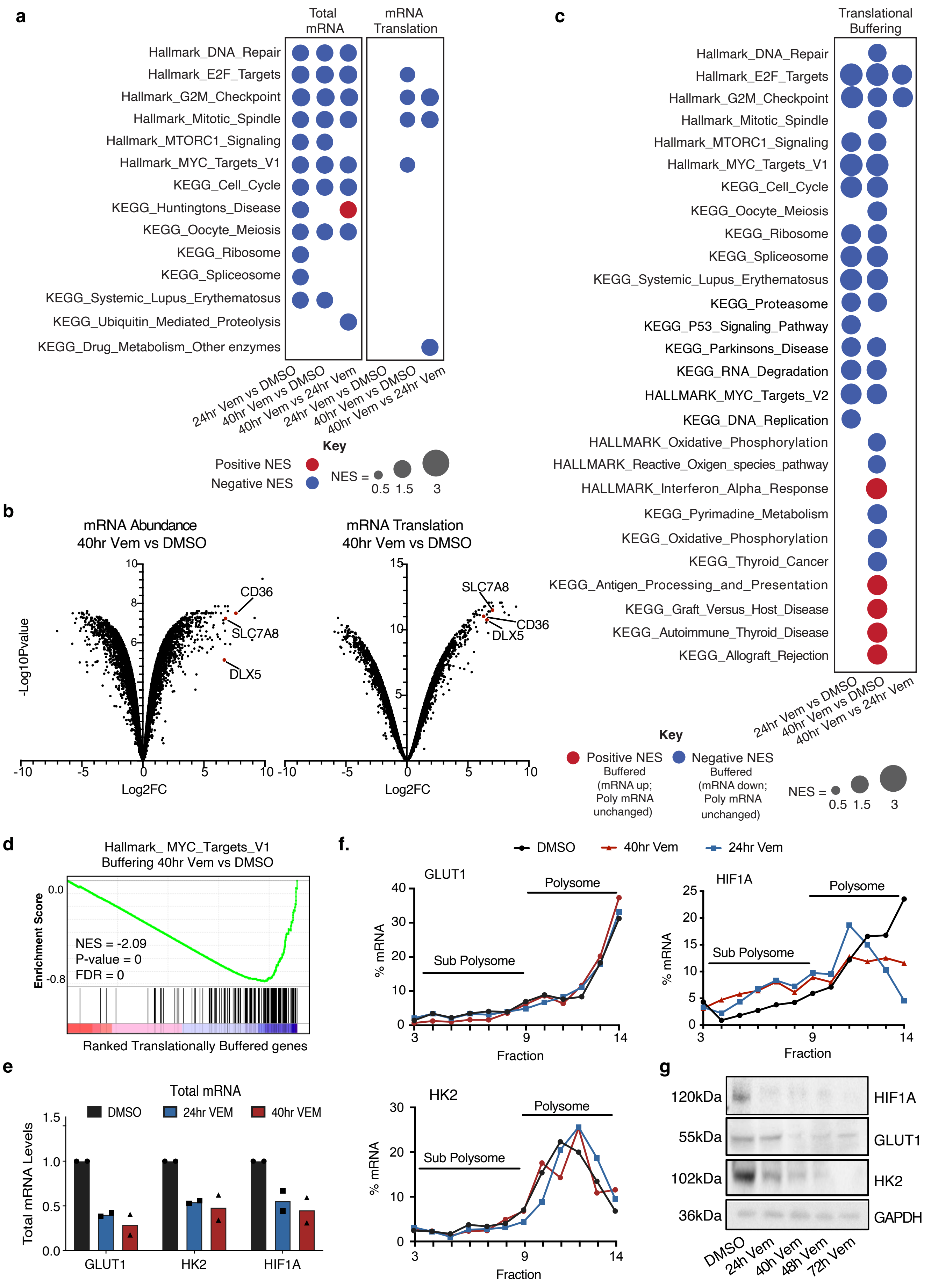
a
Total mRNA
mRNA Translation
Hallmark_DNA_Repair
Hallmark_E2F_Targets
Hallmark_G2M_Checkpoint
Hallmark_Mitotic_Spindle
Hallmark_MTORC1_Signaling
Hallmark_MYC_Targets_V1
KEGG_Cell_Cycle
KEGG_Huntingtons_Disease
KEGG_Oocyte_Meiosis
KEGG_Ribosome
KEGG_Spliceosome
KEGG_Systemic_Lupus_Erythematosus
KEGG_Ubiquitin_Mediated_Proteolysis
KEGG_Drug_Metabolism_Other enzymes
24hr Vem vs DMSO
40hr Vem vs DMSO
40hr Vem vs 24hr Vem
24hr Vem vs DMSO
40hr Vem vs DMSO
40hr Vem vs 24hr Vem
Key
Positive NES
Negative NES
NES = 0.5 1.5 3
b
mRNA Abundance 40hr Vem vs DMSO
mRNA Translation 40hr Vem vs DMSO
-Log10Pvalue
Log2FC
CD36
SLC7A8
DLX5
c
Translational Buffering
Hallmark_DNA_Repair
Hallmark_E2F_Targets
Hallmark_G2M_Checkpoint
Hallmark_Mitotic_Spindle
Hallmark_MTORC1_Signaling
Hallmark_MYC_Targets_V1
KEGG_Cell_Cycle
KEGG_Oocyte_Meiosis
KEGG_Ribosome
KEGG_Spliceosome
KEGG_Systemic_Lupus_Erythematosus
KEGG_Proteasome
KEGG_P53_Signaling_Pathway
KEGG_Parkinsons_Disease
KEGG_RNA_Degradation
HALLMARK_MYC_Targets_V2
KEGG_DNA_Replication
HALLMARK_Oxidative_Phosphorylation
HALLMARK_Reactive_Oxigen_species_pathway
HALLMARK_Interferon_Alpha_Response
KEGG_Pyrimadine_Metabolism
KEGG_Oxidative_Phosphorylation
KEGG_Thyroid_Cancer
KEGG_Antigen_Processing_and_Presentation
KEGG_Graft_Versus_Host_Disease
KEGG_Autoimmune_Thyroid_Disease
KEGG_Allograft_Rejection
24hr Vem vs DMSO
40hr Vem vs DMSO
40hr Vem vs 24hr Vem
Key
Positive NES Buffered (mRNA up; Poly mRNA unchanged)
Negative NES Buffered (mRNA down; Poly mRNA unchanged)
NES = 0.5 1.5 3
d
Hallmark_ MYC_Targets_V1
Buffering 40hr Vem vs DMSO
Enrichment Score
NES = -2.09
P-value = 0
FDR = 0
Ranked Translationally Buffered genes
e
Total mRNA
DMSO 24hr VEM 40hr VEM
Total mRNA Levels
GLUT1 HK2 HIF1A
f.
DMSO 40hr Vem 24hr Vem
GLUT1
HIF1A
HK2
Sub Polysome
Polysome
% mRNA
Fraction
g
120kDa HIF1A
55kDa GLUT1
102kDa HK2
36kDa GAPDH
DMSO 24h Vem 40h Vem 48h Vem 72h Vem

Supplementary Figure 3. BRAFi induces transcriptional and translational reprogramming of metabolism in BRAF^{V600} melanoma cells

A. Significantly enriched pathways for the indicated modes of gene expression were identified using gene set enrichment analysis (GSEA; https://www.gsea-msigdb.org/gsea/index.jsp; corrected for multiple comparisons using false discovery rate (FDR) < 0.1). Data are expressed as normalized enrichment score (NES). Source data are provided in Supplementary Data 6. **B.** Volcano plots of transcriptome-wide changes in total mRNA levels and polysome-bound mRNA (poly-mRNA; translation) identified using anota2seq following 40hr treatment with DMSO or 1μM Vem. Source data are provided in Supplementary Data 5 (n=3 biologically independent experiments). **C.** Significantly enriched pathways for the translational buffering dataset (changes in total mRNA levels not reflected in changes in poly-mRNA levels) were identified using GSEA (https://www.gsea-msigdb.org/gsea/index.jsp; corrected for multiple comparisons using FDR < 0.1). **D.** GSEA plot demonstrating enrichment of the Hallmark MYC targets (V1) gene set in the translational buffering data set. Statistical analysis was performed using the GSEA tool (https://www.gsea-msigdb.org/gsea/index.jsp; corrected for multiple comparisons using FDR < 0.1). **E.** mRNA levels of the indicated genes were determined using RT-qPCR analysis of total mRNA samples. Data are presented as mean values from n=2 biologically independent experiments. **F.** Distribution of mRNA encoding the indicated genes on a 10-50% sucrose gradient was determined using RT-qPCR following 1μM Vem treatment for the indicated time (data are representative of n=2 biologically independent experiments). **G.** Western blot analysis of the indicated proteins in whole cell lysates generated from cells treated with 1μM Vem for the indicated time (data are representative of n=2 biologically independent samples). Source data for E-G are provided as a Source Data file.

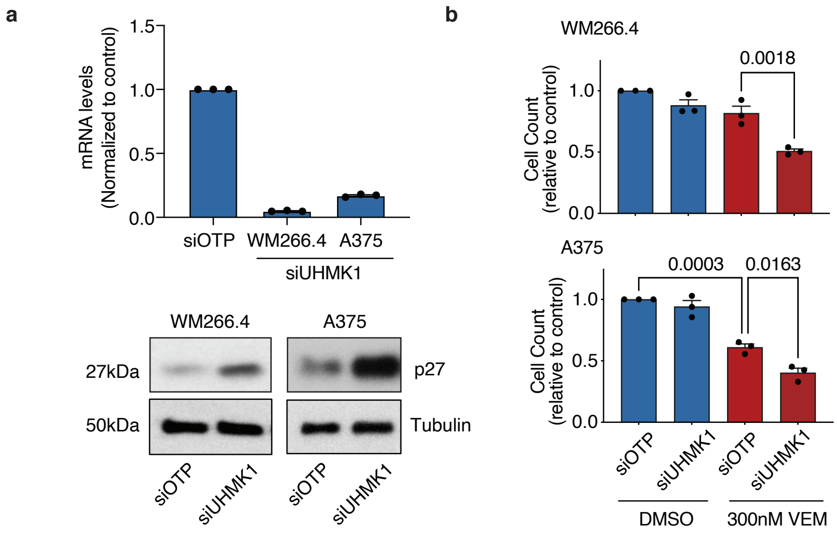

Supplementary Figure 4. Depletion of the RNA processing kinase UHMK1 sensitizes BRAF^{V600} melanoma cells to BRAF inhibition

A. WM266.4 and A375 cells were transfected with the indicated siRNA and assessed for knock down efficiency using RT-qPCR (top panel; data are presented as mean values +/- SEM, n=3 biologically independent experiments) and western blot analysis (bottom panel; data are representative of n=3 biologically independent experiments). p27 protein levels were used as a biomarker of UHMK1 knockdown, as phosphorylation by UHMK1 targets p27 for degradation (see text for details). **B.** Effect of gene knockdown and Vem treatment on cell number calculated using high content image analysis of DAPI stained cells . Data are presented as mean values +/- SEM (n=3 biologically independent experiments). Statistical significance was determined using a one-way ANOVA adjusted for multiple comparisons. Source data are provided as a Source Data file.

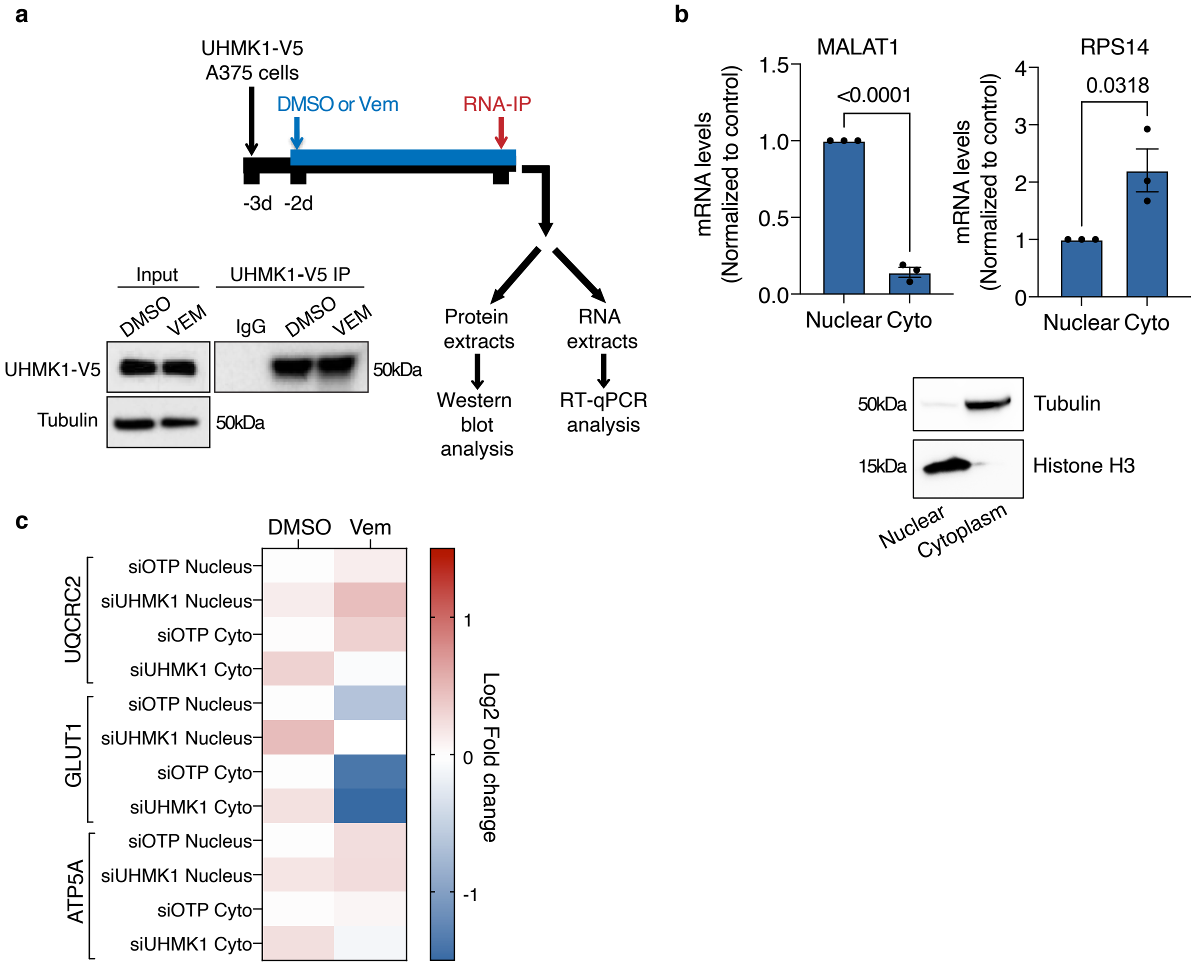

Supplementary Figure 5. Analysis of the role of RNA export in UHMK1-dependent regulation of metabolism genes following BRAF inhibition

A. A375 cells expressing UHMK1-V5 were treated with DMSO or 1μM Vem for 48hrs prior to UHMK1-V5 RNA-immunoprecipitation (RNA-IP). Schematic depicting the RNA-IP assay (top panel) and western blot verifying UHMK1-V5 immunoprecipitation (bottom panel). Data are representative of n=3 biologically independent experiments. **B.** A375 melanoma cells were transfected with the indicated siRNA and treated with DMSO or 1μM Vem as indicated for 48hrs. RNA was extracted from nuclear and cytoplasmic fractions and fractionation was verified using RT-qPCR (top panel) and western blot (bottom panel) analysis of the indicated genes and proteins. RT-qPCR data represents mean fold change +/-SEM (n=3 biologically independent experiments). Western blot data are representative of n=3 biologically independent experiments. Statistical significance was determined using an unpaired, two tailed T test. **C.** Expression of the indicated genes was determined in cells treated as in (B) using RT-qPCR analysis and Log2 fold change data relative to the DMSO siOTP control in each subcellular compartment are displayed in the heatmap (n=3 biologically independent experiments). Source data are provided as a Source Data file.

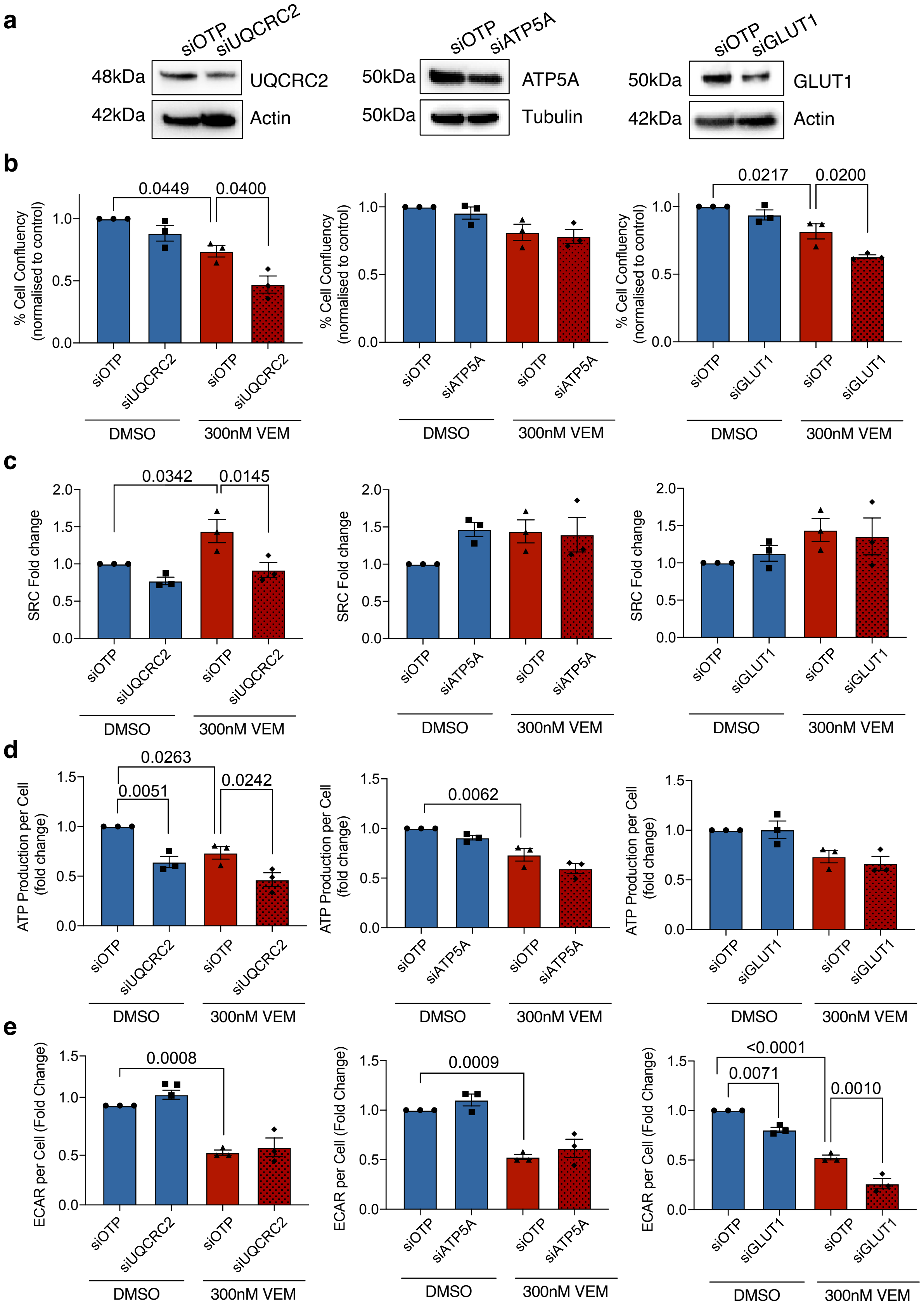

a
siOTP
siUQCRC2
48kDa
UQCRC2
42kDa
Actin
siOTP
siATP5A
50kDa
ATP5A
50kDa
Tubulin
siOTP
siGLUT1
50kDa
GLUT1
42kDa
Actin
b
% Cell Confluency (normalised to control)
0.0449
0.0400
0.0217
0.0200
c
SRC Fold change
0.0342
0.0145
d
ATP Production per Cell (fold change)
0.0263
0.0051
0.0242
0.0062
e
ECAR per Cell (Fold Change)
0.0008
0.0009
<0.0001
0.0071
0.0010
DMSO
300nM VEM

Supplementary Figure 6. Depletion of UQCRC2 and GLUT1, but not ATP5A, phenocopies UHMK1 knockdown following BRAF inhibition

A. A375 melanoma cells were transfected with the indicated siRNA and assessed for knock down efficiency using western blot analysis of the indicated proteins. Data are representative of n=2 biologically independent experiments. **B.** A375 melanoma cells were transfected with the indicated siRNA and treated with DMSO or 300nM Vem and cell proliferation was assessed by monitoring confluency over time using an Incucyte automated microscope. Average % confluency (normalised to T0) following 96hrs treatment was determined. Data represents mean fold change +/- SEM (n=3 biologically independent experiments). Statistical significance was determined using a one-way ANOVA adjusted for multiple comparisons. **C-E.** A375 cells were transfected with the indicated siRNA and treated with DMSO or 300nM Vem for 48hr. Oxygen consumption rate (OCR) and extracellular acidification rate (ECAR) was determined using Seahorse Extracellular Flux Analysis and effect of gene knockdown and Vem treatment on spare respiratory capacity (SRC) **(C)**, ATP production **(D)**, and basal glycolysis was determined **(E)**. Data is normalized to cell number and represents mean fold change +/- SEM (n=3 biologically independent experiments). Statistical significance was determined using a one-way ANOVA adjusted for multiple comparisons. Source data are provided as a Source Data file.

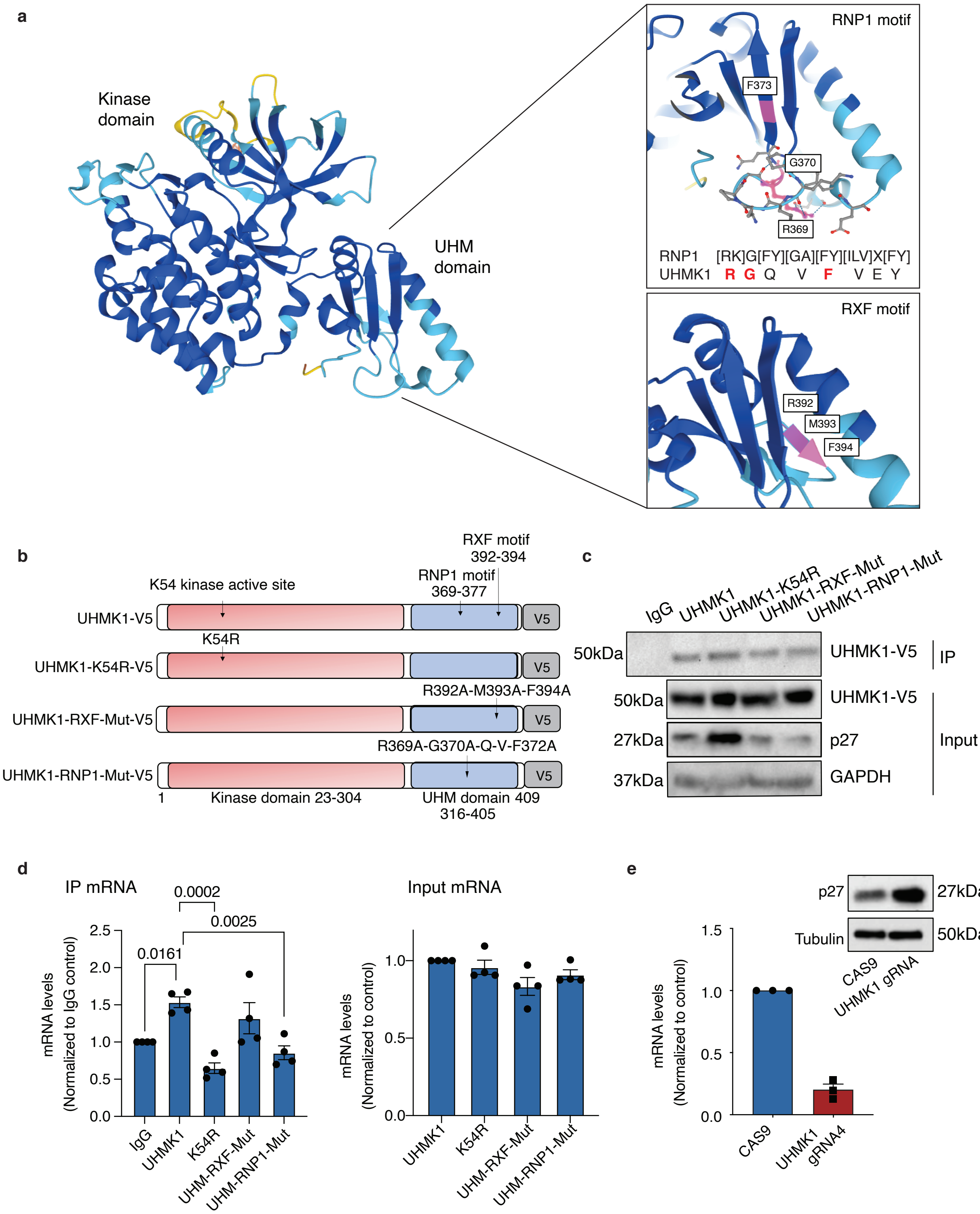
a
Kinase domain
UHM domain
RNP1 motif
F373
G370
R369
RNP1 [RK]G[FY][GA][FY][ILV]X[FY]
UHMK1 R G Q V F V E Y
RXF motif
R392
M393
F394
b
RXF motif 392-394
RNP1 motif 369-377
K54 kinase active site
UHMK1-V5
V5
K54R
UHMK1-K54R-V5
R392A-M393A-F394A
UHMK1-RXF-Mut-V5
R369A-G370A-Q-V-F372A
UHMK1-RNP1-Mut-V5
1
Kinase domain 23-304
UHM domain 409
316-405
c
IgG
UHMK1
UHMK1-K54R
UHMK1-RXF-Mut
UHMK1-RNP1-Mut
50kDa
UHMK1-V5
IP
50kDa
UHMK1-V5
27kDa
p27
Input
37kDa
GAPDH
d
IP mRNA
0.0002
0.0025
0.0161
mRNA levels
(Normalized to IgG control)
2.5
2.0
1.5
1.0
0.5
0.0
IgG
UHMK1
K54R
UHM-RXF-Mut
UHM-RNP1-Mut
Input mRNA
mRNA levels
(Normalized to control)
1.0
0.5
0.0
UHMK1
K54R
UHM-RXF-Mut
UHM-RNP1-Mut
e
p27
27kDa
Tubulin
50kDa
CAS9
UHMK1 gRNA
mRNA levels
(Normalized to control)
1.5
1.0
0.5
0.0
CAS9
UHMK1 gRNA4

Supplementary Figure 7. Structure-function analysis of UHMK1

A. Predicted structural modelling of UHMK1 protein as determined by AlphaFold (https://alphafold.ebi.ac.uk/; left panel). Conserved features of the U2AF homology motif (UHM) are shown (right panels). **B.** Schematic depicting the MSCV-UHMK1 expression constructs generated to assess the function of UHMK1 kinase activity and UHM domain. **C.** Expression and immunoprecipitation (IP) of UHMK1 mutant proteins described in (B) was verified using western blot analysis. p27 levels was used as a biomarker of UHMK1 kinase activity (see text for details; representative of n=3 biologically independent samples). **D.** Analysis of UHMK1 IP samples for β-actin mRNA was used to verify RNA processing function of the UHM domain (see text for details). Data represent mean +/- SEM of n=3 biologically independent experiments. Statistical significance was determined using a one-way ANOVA adjusted for multiple comparisons. **E.** UHMK1 was genetically inactivated using CRISPR-Cas9 and UHMK1 levels were verified using RT-qPCR and western blot analysis. Source data are provided as a Source Data file.

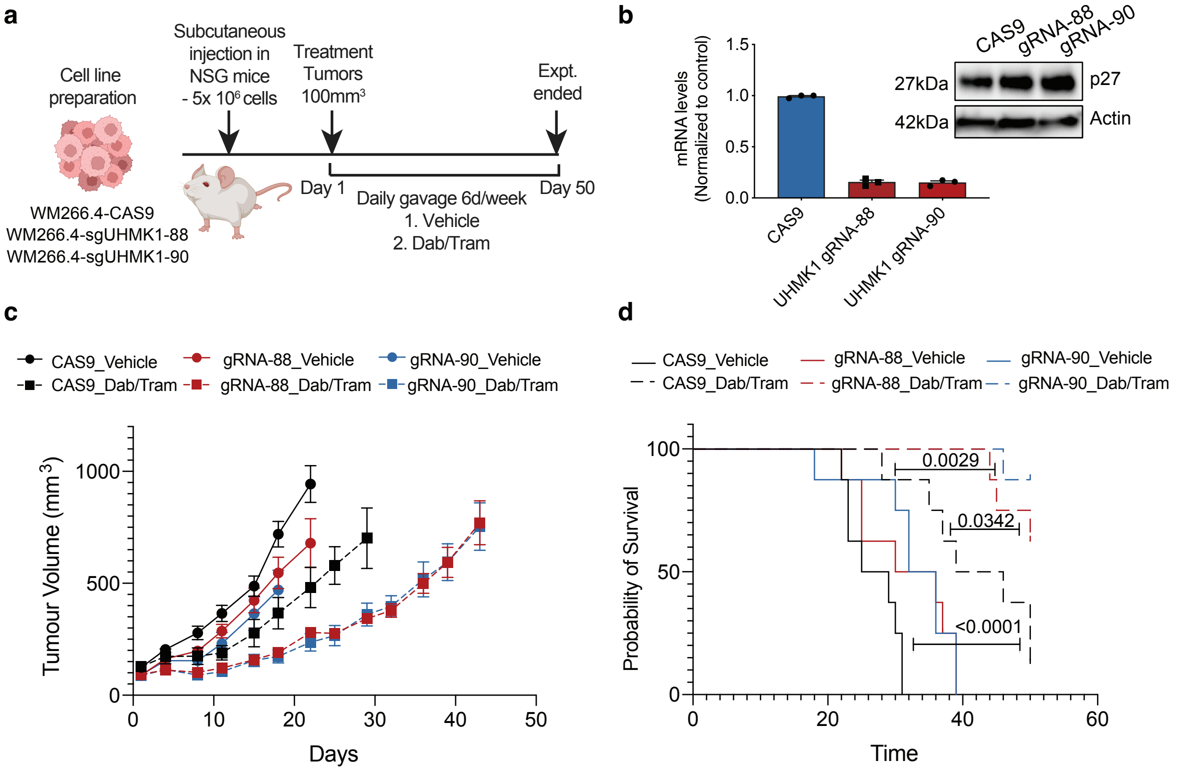

Supplementary Figure 8. Genetic inactivation of UHMK1 sensitizes WM266.4 $BRAF^{V600}$ melanoma cells to BRAF and MEK combination therapy *in vivo*

A. Schematic of the *in vivo* drug sensitivity study. **B.** UHMK1 was genetically inactivated in WM266.4 cells using CRISPR-Cas9 and UHMK1 KD was confirmed using RT-qPCR (left panel; data represent mean +/- SEM of n=3 biologically independent samples) and western blot analysis of UHMK1 target p27 (right panel; representative of n=3 biologically independent samples). **C.** Growth of WM266.4-CAS9, WM266.4-UHMK1-gRNA88 and WM266.4-UHMK1-gRNA90 tumors treated with vehicle or dabrafenib and trametinib (Dab/Tram). Data represent mean tumor growth +/- SEM of n=8 individual mice per group. **D.** Kaplan–Meier curve of data in **(C)** shows survival advantage where survival is defined as time to a tumor exceeding a volume of $1200mm^3$. Statistical significance was determined by Log-rank (Mantel-Cox) test. Source data are provided as a Source Data file.

Supplementary Table 1: Primer sequences used in the study

Gene	Direction	Sequence
ATP5A	Forward	CTTCGTTGCCACTTCCCAG
	Reverse	CCTCCGGACTGGTTCTAGG
β-actin	Forward	CTTCCTGGGCATGGAGTC
	Reverse	GGATGTCCACGTCACACTTC
GAPDH	Forward	TGCACCACCACCTGCTTAGC
	Reverse	GGCATGGACTGTGGTCATGAG
GLUT1	Forward	TCTCTGTGGGCCTTTTCGTT
	Reverse	CAGTTTCGAGAAGCCCATGAG
HK2	Forward	AAGGCAATAGGGCCTTAAAGTAGAG
	Reverse	TTCGAGGCTGCAGTGAGCTA
HIF1α	Forward	TTTACCATGCCCCAGATTCAG
	Reverse	GGTGAACTTTGTCTAGTGCTTCCA
MITF	Forward	CCGTCTCTCACTGGATTGGT
	Reverse	TACTTGGTGGGGTTTTCGAG
NONO	Forward	CATCAAGGAGGCTCGTGAGAAG
	Reverse	TGGTTGTGCAGCTCTTCCATCC
PGC1α	Forward	CTGCTAGCAAGTTTGCCTCA
	Reverse	AGTGGTGCAGTGACCAATCA
SDHB	Forward	AGAAACTGGACGGGCTCTAC
	Reverse	AACTGCAGGCCCCAGATATT
TFAM	Forward	TACCGAGGTGGTTTTCATCTG
	Reverse	AACGCTGGGCAATTCTTCTA
UHMK1	Forward	GCTGTTGATCTGTGGAGCCTA
	Reverse	TCACCACTGCTTTACTGGCA
UQCRC2	Forward	ATGTCCAAGCTGCCAAGAAC
	Reverse	GGTGGCATGTAAGAACCAGC
VDAC1	Forward	GCCCGGAAGGCAGAAGA
	Reverse	CCCTTGGTGAAGACATCCCT
