# Supplementary material for: Adaptive translational reprogramming of metabolism limits the response to targeted therapy in BRAFV600 melanoma
Source: Nat Commun. 2022 Mar 1;13:1100. doi: 10.1038/s41467-022-28705-x (PMC8888590; doi:10.1038/s41467-022-28705-x)
Supplement: Supplementary file 3 — Description of Additional Supplementary Files [file 41467_2022_28705_MOESM3_ESM.pdf]

Description of Additional Supplementary Files

Supplementary Data 1
Genome-wide siRNA screen - viability hit list. Genes below the indicated fold-change (FC) threshold are shown in sheet 1, and the corresponding enrichment analysis (EA) data are shown in sheet 2. EA performed using DAVID (https://david.ncifcrf.gov/).

Supplementary Data 2
Genome-wide siRNA screen - glycolysis hit list. Genes below the indicated fold-change (FC) threshold are shown in sheet 1, and the corresponding enrichment analysis (EA) data are shown in sheet 2. EA performed using DAVID (https://david.ncifcrf.gov/).

Supplementary Data 3
Genome-wide siRNA screen – enhancers of the BRAF inhibitor response, glycolysis and viability hit list. Genes below the indicated fold-change (FC) threshold are shown in sheet 1, and the corresponding enrichment analysis (EA) data are shown in sheet 2. EA performed using DAVID (https://david.ncifcrf.gov/).

Supplementary Data 4
RNA sequencing data for the mRNA binding, transport and translation gene set in melanoma patients on progression after treatment with MAPK pathway inhibitors. Data sourced from https://www.ncbi.nlm.nih.gov/geo/query/acc.cgi?acc=GSE65186 [24].

Supplementary Data 5
RNA sequencing data for polysome profiling analysis of A375 melanoma cells, without or without BRAF inhibition. Anota2seq was used to determine transcriptome-wide changes in mRNA abundance, translation and buffering. See methods for details.

Supplementary Data 6
Gene set enrichment analysis (GSEA) of polysome profiling RNA sequencing data. Enrichment data for the Hallmark and KEGG gene sets for the indicated comparisons are shown in separate data sheets (https://www.gsea-msigdb.org/gsea/index.jsp; NES = normalised enrichment score; See methods for details).

Supplementary Data 7
Functional annotation enrichment analysis was performed on 579 significantly buffered genes (FDR < 0.1; see Supplementary Data 5) using DAVID (https://david.ncifcrf.gov/; GO Biological Process and KEGG; P-value < 0.05).
