# Supplementary material for: Adaptive translational reprogramming of metabolism limits the response to targeted therapy in BRAFV600 melanoma
Source: Nat Commun. 2022 Mar 1;13:1100. doi: 10.1038/s41467-022-28705-x (PMC8888590; doi:10.1038/s41467-022-28705-x)
Supplement: Supplementary file 11 — Reporting summary [file 41467_2022_28705_MOESM11_ESM.pdf]

nature research

Corresponding author(s): Lorey Smith

Last updated by author(s): 23/12/21

Reporting Summary

Nature Research wishes to improve the reproducibility of the work that we publish. This form provides structure for consistency and transparency in reporting. For further information on Nature Research policies, see our Editorial Policies and the Editorial Policy Checklist.

Statistics

For all statistical analyses, confirm that the following items are present in the figure legend, table legend, main text, or Methods section.

n/a	Confirmed	
[ ]	[x]	The exact sample size (*n*) for each experimental group/condition, given as a discrete number and unit of measurement
[ ]	[x]	A statement on whether measurements were taken from distinct samples or whether the same sample was measured repeatedly
[ ]	[x]	The statistical test(s) used AND whether they are one- or two-sided<br>*Only common tests should be described solely by name; describe more complex techniques in the Methods section.*
[x]	[ ]	A description of all covariates tested
[ ]	[x]	A description of any assumptions or corrections, such as tests of normality and adjustment for multiple comparisons
[ ]	[x]	A full description of the statistical parameters including central tendency (e.g. means) or other basic estimates (e.g. regression coefficient) AND variation (e.g. standard deviation) or associated estimates of uncertainty (e.g. confidence intervals)
[ ]	[x]	For null hypothesis testing, the test statistic (e.g. *F*, *t*, *r*) with confidence intervals, effect sizes, degrees of freedom and *P* value noted<br>*Give P values as exact values whenever suitable.*
[x]	[ ]	For Bayesian analysis, information on the choice of priors and Markov chain Monte Carlo settings
[x]	[ ]	For hierarchical and complex designs, identification of the appropriate level for tests and full reporting of outcomes
[ ]	[x]	Estimates of effect sizes (e.g. Cohen's *d*, Pearson's *r*), indicating how they were calculated

Our web collection on statistics for biologists contains articles on many of the points above.

Software and code

Policy information about availability of computer code

Data collection	All data in this study was collected via commercial software as described in detail in the appropriate Methods sections.
Data analysis	All data in this study were analysed via commercial code and/ or publicly available R-packages, including: the Limma R package (v3.38.3), anota2seq (v1.4.2), GSEA software (v3.0)(Broad Institute), the GSVA 53 package (v1.20.0) in R (v3.3.2), Cytoscape (v3.8.2), DAVID (v6.8), with details of their specific use described in the appropriate Methods section.

For manuscripts utilizing custom algorithms or software that are central to the research but not yet described in published literature, software must be made available to editors and reviewers. We strongly encourage code deposition in a community repository (e.g. GitHub). See the Nature Research guidelines for submitting code & software for further information.

Data

Policy information about availability of data

All manuscripts must include a data availability statement. This statement should provide the following information, where applicable:

- Accession codes, unique identifiers, or web links for publicly available datasets
- A list of figures that have associated raw data
- A description of any restrictions on data availability

The primary siRNA screen data set is available at PubChem under the accession number AID: 1508588. The secondary deconvolution screen data set is available at PubChem under the accession number AID: 1508587. The RNAseq data generated in this study have been deposited in the GEO database under accession code GSE190071. The patient data used in this study are available in the GEO database under accession code GSE65186. The source data underlying all figures and reported analyses are provided in supplementary tables or as a source data file.

Field-specific reporting

Please select the one below that is the best fit for your research. If you are not sure, read the appropriate sections before making your selection.

- [x] Life sciences
- [ ] Behavioural & social sciences
- [ ] Ecological, evolutionary & environmental sciences

For a reference copy of the document with all sections, see nature.com/documents/nr-reporting-summary-flat.pdf

Life sciences study design

All studies must disclose on these points even when the disclosure is negative.

Sample size	No sample size calculations were performed for the experiments reported in this study. For animal studies, 9 biological replicate animals were used per treatment group based on previous experiments using this model that achieved appropriate statistical power with this sample size. Three independent biological experiments were performed for all experiments, with the exception of polysome profiling validation experiments (Figure 2 and S3) and polysome protein precipitation experiments (Figure 6). Each independent experiment contained appropriate technical replication, as determined by the standard deviation across technical replicates. We included more technical replicates to determine the mean from assays with higher intrinsic variation, for example Seahorse assays which assess metabolism in live cells, in real time, and electron acceptor rescue studies.
Data exclusions	The following data exclusions were made:<br>- One out four biological replicates for the polysome profiling analysis was excluded due to high variability between this sample and all other biological replicate samples of the same condition. Inclusion of this data point would have lead to data misinterpretation and was therefore removed from the analysis completely. The data and all analyses presented in the manuscript is from 3 independent biological replicates thus still provides adequate statistical power for analysis and ensures reproducibility of the observations across independent measurements. Exclusion criteria was not predetermined, but assessed from MDS and PCA plots.<br>- Seahorse assays were performed as 4-5 technical replicates in each independent biological replicate experiment. In some experiments, technical replicates were omitted from the analysis if they were extreme outliers due to the nature of these experiments - dynamic metabolic measurements in live cells, in real time.
Replication	We have used biological (not technical) replicates in this study to demonstrate statistical significance (by a defined methodology). We have made efforts to clearly describe in the manuscript text, relevant Methods sections and Figure Legends precisely how we chose biological replicates (i.e. animal numbers or independent repeats of in vitro experiments). For example, for the western blot assays and proliferation assays, we have shown representative data in the main figure but we have performed at least three independent experiments, unless otherwise stated.
Randomization	For mouse experiments, once tumours reached an average volume of 100mm3, mice were randomized into groups of 9 for therapy studies. For the genome wide screen, controls were randomized across alternating wells, on 2 different regions of each plate to minimize plate location effects. For tissue culture experiments, treatments were not randomized but performed in predetermined wells/dishes following a treatment layout. For these experiments, randomization was not applicable and predetermined plate layouts were essential to appropriately perform the experiments.
Blinding	For all tissue culture experiments, investigators were not blinded. Due to complex siRNA and CRISPR genetic modifications, together with drug treatments, a single investigator was responsible for all steps of the experiment and therefore could not be blind to the various conditions in each well. However, for in vivo experiments, technicians responsible for tumour measurements and subsequent statistical analyses were blinded to the nature of the experimental groups. Only the chief investigator, Dr. Lorey Smith, who did not participate in the experiments beyond generation of cell lines, knew the identity of the experimental groups.

Reporting for specific materials, systems and methods

We require information from authors about some types of materials, experimental systems and methods used in many studies. Here, indicate whether each material, system or method listed is relevant to your study. If you are not sure if a list item applies to your research, read the appropriate section before selecting a response.

Materials & experimental systems

n/a	Involved in the study	
[ ]	[x]	Antibodies
[ ]	[x]	Eukaryotic cell lines
[x]	[ ]	Palaeontology and archaeology
[ ]	[x]	Animals and other organisms
[x]	[ ]	Human research participants
[x]	[ ]	Clinical data
[x]	[ ]	Dual use research of concern

Methods

n/a	Involved in the study	
[x]	[ ]	ChIP-seq
[x]	[ ]	Flow cytometry
[x]	[ ]	MRI-based neuroimaging

Antibodies

Antibodies used	The following antibodies were used in the study: beta-actin-HRP, Sigma A3854 (1:10,000); ERK (p44/42-MAPK), Cell Signaling Technology (CST) 9102 (1:1000), phospho-ERK (p44/42-MAPK; Thr202/Tyr204), CST 9101 (1:1000); GAPDH-HRP, CST 3683 (1:3000); GLUT1, US Biologicals, G3900-0J (1:500); HIF1alpha, AB2185 (1:1000); HK2, CST 1206 (1:1000); OXPHOS antibody cocktail, AbCam AB110413 (1:1000); p27, BD transduction 610242 (1:1000); RPS6, CST 2217 (1:1000); RPL11, Invitrogen 373000 (1:1000); alpha-Tubulin, Sigma T5168 (1:10,000); V5, CST 13202 (1:1000); VDAC1, CST 4661 (1:1000).
Validation	All antibodies used in this study have been verified as per manufacturer's validation statements or profiled to determine their sensitivity, specificity and range of reactivity in the assays used.

Eukaryotic cell lines

Policy information about cell lines

Cell line source(s)	WM266.4, A375, MALME3, SKMEL28, IPC298, D04M1, and HEK-293T cell lines were obtained from ATCC.
Authentication	All cell lines were authenticated using STR profiling, using a polymerase chain reaction (PCR) based short tandem repeat (STR) analysis using six STR loci. CRISPR-knock out clones were also verified after single cell sorting and expansion to confirm their identity.
Mycoplasma contamination	Mycoplasma testing was routinely performed by PCR. Cell lines were only used upon confirmation of mycoplasma negative status.
Commonly misidentified lines (See ICLAC register)	No commonly misidentified cell lines were used in this study.

Animals and other organisms

Policy information about studies involving animals; ARRIVE guidelines recommended for reporting animal research

Laboratory animals	Mus musculus, NOD-Scid interleukin 2 receptor gamma chain null (NSG), female and male, 6-7 weeks old
Wild animals	The study did not involve wild animals.
Field-collected samples	Study did not involve samples collected from the field.
Ethics oversight	All animal studies were performed according to protocols approved by the Animal Ethics Committee of Peter MacCallum Cancer Centre and in accordance with the National Health and Medical Research Council Australian code for the care and use of animals for scientific purposes, 8th Edition, 2013.

Note that full information on the approval of the study protocol must also be provided in the manuscript.
